# In Vitro Activity of Novel Antifungal Olorofim against Filamentous Fungi and Comparison to Eight Other Antifungal Agents

**DOI:** 10.3390/jof7050378

**Published:** 2021-05-12

**Authors:** Ourania Georgacopoulos, Natalie S. Nunnally, Eric M. Ransom, Derek Law, Mike Birch, Shawn R. Lockhart, Elizabeth L. Berkow

**Affiliations:** 1 Mycotic Diseases Branch, Centers for Disease Control and Prevention, Atlanta, GA 30333, USA; nua5@cdc.gov (N.S.N.); gyi2@cdc.gov (S.R.L.); kuu4@cdc.gov (E.L.B.); 2 Association of Public Health Laboratories, Silver Springs, MD 20910, USA; ericmransom@wustl.edu; 3 F2G Ltd., Manchester M30 0LX, UK; dlaw@F2G.com (D.L.); mbirch@F2G.com (M.B.)

**Keywords:** olorofim, F901318, filamentous fungi, antifungal agents, *Aspergillus fumigatus*, *Fusarium* spp., dark molds, multidrug resistance

## Abstract

Olorofim is a novel antifungal drug that belongs to the orotomide drug class which inhibits fungal dihydroorotate dehydrogenase (DHODH), thus halting pyrimidine biosynthesis and ultimately DNA synthesis, cell growth and division. It is being developed at a time when many invasive fungal infections exhibit antifungal resistance or have limited treatment options. The goal of this study was to evaluate the in vitro effectiveness of olorofim against a large collection of recently isolated, clinically relevant American mold isolates. In vitro antifungal activity was determined for 246 azole-susceptible *Aspergillus fumigatus* isolates, five *A. fumigatus* with TR_34_/L98H-mediated resistance, 19 *Rhizopus* species isolates, 21 *Fusarium* species isolates, and one isolate each of six other species of molds. Olorofim minimum inhibitory concentrations (MICs) were compared to antifungal susceptibility testing profiles for amphotericin B, anidulafungin, caspofungin, isavuconazole, itraconazole, micafungin, posaconazole, and voriconazole. Olorofim MICs were significantly lower than those of the echinocandin and azole drug classes and amphotericin B. *A. fumigatus* wild type and resistant isolates shared the same MIC50 = 0.008 μg/mL. In non-*Aspergillus* susceptible isolates (MIC ≤ 2 μg/mL), the geometric mean (GM) MIC to olorofim was 0.54 μg/mL with a range of 0.015–2 μg/mL. Olorofim had no antifungal activity (MIC ≥ 2 μg/mL) against 10% of the collection (31 in 297), including some isolates from *Rhizopus* spp. and *Fusarium* spp. Olorofim showed promising activity against *A. fumigatus* and other molds regardless of acquired azole resistance.

## 1. Introduction

Invasive fungal infections (IFI) are increasing in prevalence globally in parallel with the increase in antifungal drug resistance, posing a serious challenge for healthcare providers [1,2,3]. Advances in immunosuppressive therapies have resulted in a growing population of immunocompromised patients who are vulnerable to fungal infections including individuals with HIV, cancer patients, organ transplant recipients, stem cell transplant recipients, and people with long-term hospitalizations [4]. Several pathogenic mold species have exhibited increasing antifungal resistance, chief among them *Aspergillus fumigatus* [5]. Invasive aspergillosis is the predominant invasive mold infection in patients, with *Aspergillus* increasingly resistant to first line triazole antifungals [6,7]. Other opportunistic molds, such as *Rhizopus*, *Fusarium,* and some dematiaceous mold species, also contribute to the burden of IFI in the healthcare setting and are increasingly refractory to available antifungal therapies [8].

Azoles, echinocandins, and the polyene amphotericin B are the only antifungals currently approved to treat invasive mold infections (IMIs). Although amphotericin B is often an effective therapy, it is associated with many adverse side effects including infusion-related reactions and nephrotoxicity [9]. Azole use can have side effects as well. Azoles are not 100% selective for binding to fungal targets (they can also bind to human CYP450) which can cause drug-drug interactions [10]. Long-term azole use can lead to acquired drug resistance, which is a concern as it leads to higher rates of treatment failure and longer hospital stays [11]. In addition to increasing azole resistance due to broader use of azoles in medicine, the use of azole compounds as agricultural fungicides has led to an increase in azole-resistant *A. fumigatus*, especially in Europe [11,12,13,14,15]. Challenges also exist in treating other rare or unusual mold infections. The paucity of new drug development in the antifungal pipeline has limited the treatment options for rare and difficult to treat molds such as the Mucorales, *Fusarium* and dematiaceous molds [16,17]. New drug development is needed to help combat these infections.

Olorofim (F2G Limited, Manchester, UK) is the first antifungal in a new drug class known as orotomides. Olorofim inhibits fungal dihydroorotate dehydrogenase (DHODH), halting pyrimidine biosynthesis and ultimately impacting DNA synthesis, cell growth and division. DHODH is a unique drug target rendering it less likely to be impacted by other acquired resistance mechanisms [18]. The fungal DHODH target differs significantly from human DHODH, minimizing target-based drug toxicity [19].

Olorofim displays antifungal activity against numerous species of molds including *Penicillium* spp., *Coccidioides* spp., *Histoplasma capsulatum*, *Blastomyces dermatitidis*, *Fusarium* spp., *Scedosporium* spp., *Lomentospora prolificans*, *Scopulariopsis brevicaulis*, and *Aspergillus* spp. [18,19,20,21,22,23,24,25,26,27,28]. The FDA has granted olorofim breakthrough therapy designation and orphan drug designation for treatment of invasive aspergillosis as well as for infections due to Lomentospora/Scedosporium, Scopulariopsis, and central nervous system (CNS) coccidioidomycosis. An open-label single-arm phase 2b study for treatment of invasive fungal infections in patients who lack treatment options is underway (ClinicalTrials.gov Identifier: NCT03583164) [29].

In this study we assessed the in vitro efficacy of olorofim compared to MIC values of traditional antifungals using the Clinical and Laboratory Standards Institute (CLSI) reference method for antifungal susceptibility testing. Olorofim, azoles, echinocandins, and polyenes were tested against 297 contemporary mold isolates, including those which are refractory to currently available antifungal treatments.

## 2. Materials and Methods

A total of 297 mold isolates were tested, including azole-susceptible *A. fumigatus* (*n* = 246), azole-resistant *A. fumigatus* with the TR_34_/L98H (*n* = 3) and TR34/L98H/S297T/F495I (*n* = 2) mutations, *Rhizopus microsporus* (*n* = 3), *Rhizopus oryzae* (*n* = 16), *Fusarium chlamydosporum* (*n* = 1), *Fusarium dimerum* (*n* = 2), *Fusarium moniliforme* (*n* = 1), *Fusarium oxysporum* (*n* = 5), *Fusarium verticillioides* (*n* = 1), *Fusarium solani* species complex (*n* = 11), and rare and unusual mold species including *Phialemonium curvatum* (*n* = 1), *Phaeoacremonium parasticum* (*n* = 1)*, Sarocladium kiliense* (*n* = 1), *Ramularia* species (*n* = 1), *Metarrhizium anisopliae* (*n* = 1), and *Pleurostomophora richardsiae* (*n* = 1). All mold isolates were received at CDC between 1998 and 2019 as part of ongoing surveillance and routine diagnostic testing and come from both clinical and environmental sources. All isolates were clinical except for 12 *R. microsporus* and *R. oryzae* isolates which came from environmental sources, and 90% of isolates were received between 2017 and 2019.

*A. fumigatus* isolates were screened for itraconazole, posaconazole, and voriconazole resistance using a plate assay as described [30]. Isolates with breakthrough growth on the plate were confirmed as resistant to azoles by standard broth microdilution according to the Clinical and Laboratory Standards Institute (CLSI) document M-38 [31]. Epidemiologic cutoff values for *A. fumigatus* of ≥2 μg/mL for itraconazole and/or ≥1 μg/mL for voriconazole were used to indicate reduced antifungal activity. Mutations in the *Cyp51A* gene were confirmed through gene sequencing as previously described [32].

Susceptibility testing of mold isolates against olorofim followed the methods described in CLSI reference standard M38-A2 (M38 Reference). F901318 (olorofim) powder was donated by F2G Limited (Manchester, UK). Dilutions of olorofim were prepared using DMSO and the concentration range set to (0.0001–2 μg/mL) in 2-fold serial dilutions. Olorofim drug plates were prepared using 96-well polystyrene round-bottom microwell plates (Thermo Scientific, Item ID# 262162) and 100 μL of synthetic medium RPMI-1640 was added to the plates before dispensing the antifungal. The HP D300e Digital Dispenser, HP Dispensing Software and HP T8+ and HP D4+ Dispensing cassettes were used to dispense the predetermined dosages of olorofim [33]. Drug plates were prepared ahead of time and stored at −80 °C until day of use, when they were removed from the freezer and allowed to thaw in a 37 °C incubator.

Isolates were cultured onto Sabouraud Dextrose (SabDex) agar slants and incubated for 2–7 days at 35 °C. The suspension was prepared by adding 1 mL of Tween 20 (2%, prepared in molecular grade H_2_O) to each agar slant. The solution was drawn off and heavier particles allowed to settle. Absorbance was determined by measuring the optical density at 530 nm (OD530) using a spectrophotometer and adjusted to the desired range with sterile water as follows: OD_530_ of 0.09 to 0.13 for *Aspergillus* and dematiaceous spp., and OD_530_ of 0.15–0.17 for *Fusarium* and *Rhizopus* spp. For antifungal susceptibility testing the adjusted suspension was diluted 1:50 in RPMI-1640 broth and 0.1 mL of the diluted inoculum added to each well. A growth control well containing no antifungal agent was included, in addition to a well containing only the medium and nuclease free water as a negative control. Quality control isolates for each species tested are listed in Table 1.

Olorofim antifungal activity was compared with results for other drugs from antifungal susceptibility testing using custom frozen panels from TREK Diagnostics (Thermo Fisher Scientific, Oakwood Village, OH, USA), which included anidulafungin, caspofungin, isavuconazole, itraconazole, micafungin, posaconazole, and voriconazole. Amphotericin B susceptibility was determined using Etest (bioMérieux, France). MICs were read for amphotericin B and the azoles, whereas the MEC was read for the echinocandins as specified in the CLSI’s M38 [31]. Endpoints were recorded at 24 h for *Rhizopus* spp. and 48 h for *A. fumigatus*, *Fusarium* spp., *Phialemonium curvatum*, *Phaeoacremonium parasticum, Sarocladium kiliense*, *Ramularia* species, *Metarrhizium anisopliae*, and *Pleurostomophora richardsiae.*

## 3. Results

### 3.1. CLSI Reference Method MIC Results for Olorofim, Azoles, Echinocandins and Amphotericin B against Molds

#### 3.1.1. Olorofim

**Figure 1:**
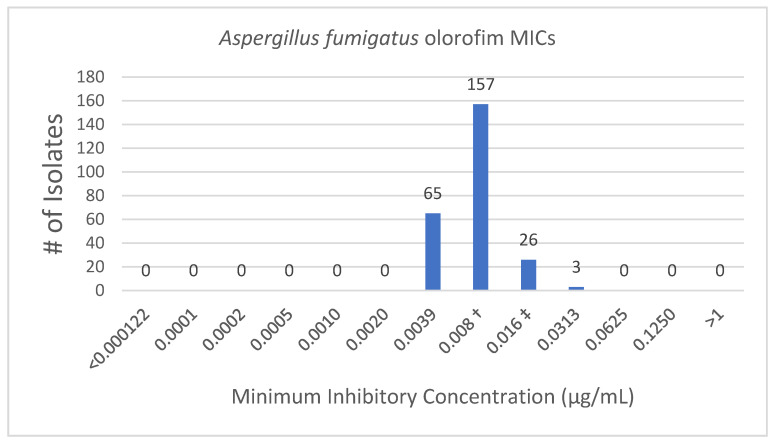
Distribution of olorofim MICs for Aspergillus fumigatus isolates. † MIC 50; ‡ MIC 90.

Olorofim MIC results for each species tested are listed in Table 2. Olorofim showed consistent antifungal activity when tested against azole-susceptible *A. fumigatus* isolates (MIC_50_ = 0.008 μg/mL). All *A. fumigatus* isolates fell within a one to two dilution range of the MIC_50_ (0.008 μg/mL) (Figure 1). The five azole-resistant *A. fumigatus* isolates with *Cyp51A*-associated point mutations had MIC values of 0.008 μg/mL, the same as the MIC_50_ of azole-susceptible *A. fumigatus* isolates.

Olorofim showed variable antifungal activity amongst *Fusarium* spp. isolates. Olorofim showed no antifungal activity against *F. chlamydosporum* in the range tested (MIC > 2 μg/mL). *F. dimerum* and *F. solani* species complex MICs were in the upper range of tested concentrations or showed no antifungal effect (MIC range 2 to >2 μg/mL). Olorofim had a wide range of antifungal activity against *F. oxysporum* (MIC = 0.12 to >2 μg/mL). *F. moniliforme* and *F. verticilloides* had low olorofim MICs compared to other *Fusarium* spp. tested (MIC = 0.03 and 0.50 μg/mL, respectively)**.** *Phialemonium curvatum* and *Phaeoacremonium parasiticum* were in the upper range tested (MIC = 2 μg/mL), while *Sarocladium kiliense* (MIC = 0.5 μg/mL), *Ramularia* spp. (MIC = 0.015 μg/mL), *Metarrhizium anisopliae* (MIC = 0.5 μg/mL)*,* and *Pleurostomophora richardsiae* (MIC = 0.06 μg/mL) all had low olorofim MIC values. Olorofim did not show in vitro inhibitory activity against *Rhizopus microsporus* or *Rhizopus oryzae* in the range tested, in agreement with other studies that show olorofim is ineffective against members of the Mucorales group.

#### 3.1.2. Azoles

The collection was tested against the following azoles: isavuconazole, itraconazole, posaconazole, and voriconazole. Aside from *A. fumigatus*, voriconazole showed little antifungal activity against most isolates in this collection although the MICs to *R. microsporus* and *A. fumigatus* with mutations in TR_34_/L98H and TR_34_/L98H/S297T/F495I were variable. For itraconazole, isolates of *R. oryzae* had variable MICs (range 0.03 μg/mL to >16 μg/mL) while all other species had only high MICs. *R. microsporus*, *F. verticillioides*, and *Ramularia* species all had low MICs to isavuconazole (≤1 μg/mL), however isavuconazole was not as effective against the remaining species, all of which had MIC in the upper range (≥8 μg/mL). Consistent with the results for itraconazole, *R. oryzae* had variable MICs to isavuconazole (range 0.25 μg/mL to >8 μg/mL). For posaconazole, *R. microsporus*, *F. verticilloides*, *P. curvatum*, *P. parasiticum*, and *Ramularia* species all had low MICs (≤0.5 μg/mL). All other species had high MICs to posaconazole (≥2 μg/mL) except for *R. oryzae*, which had variable MICs (range 0.03 μg/mL to >16 μg/mL).

#### 3.1.3. Echinocandins

The collection was tested against anidulafungin, caspofungin, and micafungin. Minimal effective concentrations (MECs) were high for all three echinocandins, ≥2 μg/mL for *R. oryzae*, *F. chlamydosporum*, *F. dimerum*, *F. verticilloides*, *P. curvatum,* and *P. parasiticum*. For *S. kiliense*, *R. microsporus*, *F. oxysporum,* and *Fusarium solani* species complex echinocandin MECs were variable (range 0.06 μg/mL to >16 μg/mL). The remainder of the collection displayed low MECs to the echinocandins (≤0.125 μg/mL).

#### 3.1.4. Amphotericin B

Amphotericin B was more effective than either the azoles or echinocandins. *Rhizopus microsporus*, *F. moniliforme*, *P. curvatum*, *P. parasiticum*, and *P. richardsiae* all exhibited low MICs to amphotericin B (≤0.5 μg/mL). *Fusarium chlamydosporum*, *F. dimerum*, *F. verticilloides, S. kiliense*, *Ramularia* species, and *Metarrhizium anisopliae* all exhibited high MICs to amphotericin B (≥2 μg/mL). The MIC values for *R. oryzae*, *F. oxysporum,* and *F. solani* species complex were variable (range 0.25 μg/mL to >32 μg/mL).

## 4. Discussion

Our study corroborates previous findings showing the in vitro efficacy of olorofim against *A. fumigatus* and provides new data on rare and unusual molds. Previous studies exploring efficacy of olorofim against *Aspergillus* spp. found similar MIC ranges and susceptibility patterns. In Jørgensen et al., 235 *A. fumigatus* isolates were tested using the EUCAST method, resulting in a geometric mean MIC of 0.037 μg/mL, which is consistent with our results despite the differences in testing methodology [20,34]. Buil et al. tested 10 *A. fumigatus* WT isolates and reported an olorofim MIC_50_ of 0.06 μg/mL, the olorofim MICs of isolates with the TR_34_/L98H mutation were in the same range as the WT isolates (0.031–0.125 mg/L) [18]. As olorofim has a completely different target to the azoles, cross-resistance would not be expected. Consistent with this, an in-silico model predicts olorofim to possess a low probability of developing resistance in *A. fumigatus* [35]. A limitation of this study is the small sample size (*n* = 5) of *A. fumigatus* isolates with the TR_34_/L98H mutation that were tested. The addition of our large collection of contemporary *A. fumigatus* isolates contributes to our knowledge of the wild type MIC range of this species against olorofim and will help with the establishment of epidemiological cutoff values. In addition, it shows that US isolates of *A. fumigatus* have similar susceptibility to olorofim as European isolates.

Jørgensen et al. used the EUCAST method to test olorofim susceptibility against different species of *Fusarium* and found *F. dimerum* and *F. solani* to have MICs > 1μg/mL [20]. Our study tested multiple species of *Fusarium* and found elevated MICs of ≥ 2 μg/mL for all species except *F. verticillioides* (MIC 0.50 μg/mL) and an isolate of *F. oxysporum*. Wiederhold et al. similarly found *F. verticillioides* had low MIC values with a range of 0.03–0.125 μg/mL, indicating olorofim is likely to be active against *F. verticillioides,* but in many cases Fusarium are not identified to species so this observation may not be clinically relevant [36]. It was also shown that olorofim activity against *Fusarium* is endpoint dependent, and that using a 50% inhibition endpoint results in lower MICs [36].

*Rhizopus* species remain difficult to treat and can have very high MIC values to amphotericin B and posaconazole [17,37,38,39]. Isavuconazole is the only antifungal with a US Food and Drug Administration indication for use against Mucorales. A recent study looked at *R. microsporus* susceptibility to olorofim and found no antifungal activity (*n* = 4, MIC ≥ 1) [20]. The lack of antifungal activity of olorofim against Mucorales species can be explained by differences in the DHODH drug target. Mucorales’ DHODH is distantly related to the DHODH of susceptible fungal species [19]. Dematiaceous and other rare molds can cause serious infections and are difficult to treat as many antifungals have little activity and effective treatment information is lacking. We included several rare molds from clinical cases of fungal infection to highlight the possible range of olorofim activity beyond the most commonly seen fungal infections. The activity of olorofim cannot be generalized for these rare infections but has shown good activity for some species and warrants further investigation. In conclusion we have confirmed the efficacy of olorofim against both WT and azole-resistant *A. fumigatus* seen in other studies as well demonstrated in vitro efficacy of olorofim against several *Fusarium* spp. and rare molds.

## Figures and Tables

**Table 1:** Quality control isolates used for each fungal organism tested.

Quality Control or Reference Strain	Species Tested
*Aspergillus fumigatus* ATCC MYA-3626	*Fusarium* spp., *Phialemonium curvatum*, *Phaeoacremonium parasiticum*, *Sarocladium kiliense*, *Ramularia* sp., *Metarrhizium anisopliae* and *Pleurostomophora richardsiae*
*Candida krusei* ATCC 6258	*Rhizopus* spp.
Azole susceptible *A. fumigatus* B7698 Azole resistant *A. fumigatus* B7815 (CDC internal controls)	*A. fumigatus*

**Table 2:** MIC results of Olorofim and AFST testing using the CLSI M38-A2 Reference Method for Broth Dilution. MIC 50 and ranges listed in μg/mL. MIC is reported for species with only 1 isolate tested.

Species	Olorofim	Voriconazole	Anidulafungin	Caspofungin	Itraconazole	Isavuconazole	Posaconazole	Micafungin	Amphotericin B
*A. fumigatus* WT (*n* = 246)									
MIC 50	0.008								
Range	0.004–0.03								
*A. fumigatus* TR34/L98H (*n* = 5)									
MIC 50	0.008	2			16				
Range	0.008–0.008	0.3–2			4–>16				
*R. microsporus* (*n* = 3)									
MIC 50	>2	4	4	16	0.06	0.5	0.5	>8	0.06
Range	2–>2	0.5–4	0.25–>16	0.25–>16	0.06–0.125	0.125–1	0.06–0.125	0.25–>8	0.016–0.125
*R. oryzae* (*n* = 16)									
MIC 50	>2	4	>16	>16	0.125	4	0.3	>8	1.5
Range	>2–>2	1–8	8–>16	16–>16	0.03–>16	0.25–>8	0.03–>16	4–>8	0.3–4
*F. chlamydosporum* (*n* = 1)									
MIC 50	>2	2	>16	>16	>16	>8	>16	>8	2
Range									
*F. dimerum* (*n* = 2)									
MIC 50	>2	4	>16	>16	>16	>8	>16	>8	4
Range	2–>2	4–4	8–>16	16–>16	>16–>16	8–>8	>16–>16	2–>8	3–4
*F. moniliforme* (*n* = 1)									
MIC 50	0.03	2	0.06	0.125	>16	>8	>16	0.02	0.5
Range									
*F. oxysporum* (*n* = 5)									
MIC 50	2	16	16	16	>16	>8	>16	8	24
Range	0.12–>2	4–>16	0.125–>16	0.125–>16	>16–>16	8–>8	2–>16	0.06–>8	0.8–>32
*F. solani* (*n* = 11)									
MIC 50	>2	8	8	8	>16	>8	>16	4	>32
Range	2–>2	1–>16	1–>16	2–>16	>16–>16	>8–>8	>16–>16	0.125–>8	1.5–>32
*F. verticilloides* (*n* = 1)									
MIC 50	0.5	1	>16	>16	0.3	1	0.06	>8	>32
Range									
*M. anisopliae* (*n* = 1)									
MIC 50	0.5	1	0.125	0.06	>16	8	>16	<0.008	>32
Range									
*P. parasiticum* (*n* = 1)									
MIC 50	2	1	8	>16	>16	8	0.5	>8	0.3
Range									
*P. curvatum* (*n* = 1)									
MIC 50	2	1	8	>16	0.5	8	0.125	>8	0.125
Range									
*P. richardsiae* (*n* = 1)									
MIC 50	0.06								0.4
Range									
*Ramularia* species (*n* = 1)									
MIC 50	0.015	1	<0.008	<0.008	0.5	1	0.3	<0.008	16
Range									
*S. kiliense* (*n* = 1)									
MIC 50	0.5	2	8	0.5	>16	>8	>16	4	12
Range									

## Data Availability

Not applicable.
